# IBD Patients with Primary or Secondary Nonresponse to Ustekinumab Benefit from Dose Escalation or Reinduction

**DOI:** 10.3390/jcm13143993

**Published:** 2024-07-09

**Authors:** Filippo Vernia, Sabrina Monaco, Giovanni Latella

**Affiliations:** Division of Gastroenterology, Hepatology, and Nutrition, Department of Life, Health and Environmental Sciences, University of L’Aquila, 67100 L’Aquila, Italy; filippo.vernia1@gmail.com (F.V.); monacosa6@gmail.com (S.M.)

**Keywords:** ustekinumab, dose escalation, reinduction, IBD, inflammatory bowel disease

## Abstract

Ustekinumab is a monoclonal antibody approved for the treatment of IBD. This drug has a well-established efficacy; however, patients may not respond or lose response. The availability of other biological therapies prompts the need for comparative data between different agents to suggest first- or second-line strategies. Aim of this review is to compare the effectiveness of ustekinumab to other biologics in Crohn’s disease and ulcerative colitis, as well as report the available data on dose escalation and reinduction. A systematic electronic search of the English literature was performed up to November 2023, using Medline (PubMed), Web of Science, Scopus and the Cochrane Library. Conference proceedings were also screened. Out of 659 citations, 80 relevant articles were selected and included in the present narrative review. Head-to-head comparisons of different biological drugs are relatively scarce, mostly deriving from indirect comparison or retrospective studies. Overall available data indicate similar effectiveness in the treatment of IBD patients. Dose escalation and reinduction strategies are well documented, but the optimal treatment schedule is still to be defined. Response and remission rates vary in different studies, and a proportion of patients fail to achieve clinical and endoscopic outcomes. However, both approaches are effective and safe in nonresponders and secondary loss of response. IBD patients may benefit from dose escalation or reinduction. Both strategies prove effective in regaining response in a proportion of patients, avoiding unnecessary early switch. Head-to-head trials are still needed to determine the exact placement of this drug compared to other biologics.

## 1. Introduction

The medical treatment of IBD, Crohn’s disease (CD) and ulcerative colitis (UC) was traditionally based on conventional drugs, including 5-aminosalicylates (5-ASAs), corticosteroids and immunomodulators [1]. The availability of biologics, two decades ago, and more recently of small molecules, changed the therapeutic scenario, offering new effective therapeutic tools.

5-ASA and Salazopyrin (SASP) are moderately effective in inducing and maintaining remission in UC [2], but not in CD [3]. Thiopurines (Azathioprine and 6-Mercaptopurine) and methotrexate proved useful in IBD but their utilization as monotherapy declined following the introduction of biologics. Thiopurines, but not methotrexate [4], are presently used in association with biological therapies to reduce immunogenicity and secondary loss of response [5]. The association of Azathioprine and anti-tumor necrosis factor α (TNFα) indeed is more effective than anti-TNFα alone in UC (40% vs. 20%, *p* < 0.0019) [6], and CD (relative risk (RR) 1.23, 95% confidence interval (CI) 1.02–1.47) [7].

The introduction of anti-TNFα [8], and other biologic agents, including anti-integrins, anti-interleukins and small molecule therapy (Tofacitinib (TOF), Filgotinib and Upadacitinib (UPA)) provided effective additional therapeutic options [9]. Their efficacy was confirmed by the significant decline in overall emergency surgery rates at 5 years [10,11], although results were less impressive in severe, complicated UC [12].

Biologics, however, do not represent the golden bullet because approximately one-third of biologic-naïve patients do not respond to anti-TNFα induction and in up to 45% of cases, efficacy is lost over time [13]. A high rate of primary or secondary loss of response is also observed with all other biologics. The choice of escape strategies is thus of prime importance. The present narrative review has been focused on the therapeutic approach in patients undergoing inadequate response and loss of response following induction with ustekinumab (UST), a fully human monoclonal antibody targeting the p40 subunit of interleukin-12 and interleukin-23 [14]. IL-12 and IL-23 are secreted by activated antigen-presenting cells and induce inflammatory and immune functions including natural killer cells activation, CD4+ T-cells differentiation into the T-helper 1 and T-helper 17 induction. UST prevents IL-12 and IL-23 interaction with the surface IL-12Rb1 receptor complexes, thus reducing immune cell activation [14].

The suggested treatment schedule of UST consists of a single intravenous infusion at the dose of 6 µg/kg body weight, followed by subcutaneous (SC) 90 µg injections every 12 or 8 weeks (q12w–q8w). Available data suggest that about one-fourth of CD and UC patients are primary nonresponders, and loss of response must be expected at a rate of 20% per patient/year. Thus, before deciding to move to other classes of biologics, dose optimization, or reinduction, must be considered.

Despite being common in clinical practice, UST dose escalation and reinduction in CD and UC patients are not listed in the drug information leaflet. This paper focuses on the off-label use of UST, and analyzes the efficacy of reinduction or dose escalation in primary nonresponders and patients with secondary loss of response to favor effective clinical decision making.

## 2. Materials and Methods

A systematic electronic search of the English literature was performed up to November 2023, using Medline (PubMed), Web of Science, Scopus and the Cochrane Library. The search included a combination of Medical Subject headings (MeSH) and keywords: “IBD”, “Inflammatory Bowel Disease”, “Crohn”, “ulcerative colitis”, “inflammation”, “cytokines”, “immune system”, “biologic therapy” and “Ustekinumab”. Conference proceedings were also screened.

Two authors (F.V.; S.M.) identified relevant articles by screening the abstracts. Additional studies were selected after a manual review of the reference list of the identified studies and review articles. Any discrepancy was resolved by consensus, referring to the original articles. Out of 659 citations, 80 relevant articles were selected and included in the present narrative review.

## 3. Results

### 3.1. Ustekinumab, Registrative Trials and Treatment in Naïve CD Patients

**Figure 1:**
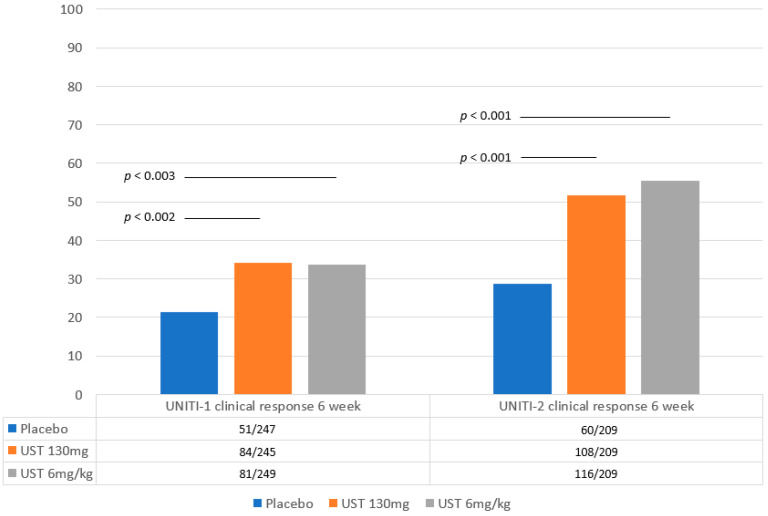
**Primary endpoints of the UNITI 1 and UNITI 2 trials.** Primary endpoints (clinical response) of induction studies UNITI 1 and UNITI 2 at 6 weeks in patients with moderate-to-severe Crohn’s disease receiving Ustekinumab or placebo [15]. Statistical significance values refer to those reported in the original papers.

**Figure 2:**
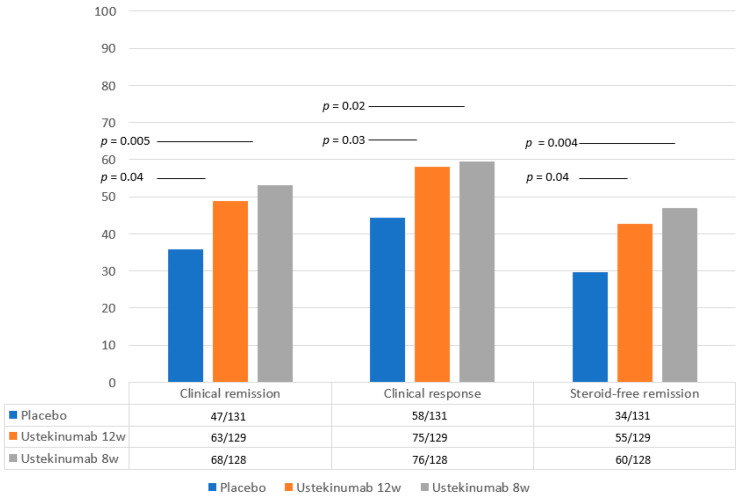
**Primary and secondary endpoints of the IM-UNITI trial.** Primary endpoint (clinical remission) and secondary endpoints (clinical response and steroid-free remission) of the maintenance study (IM-UNITI) at 22 weeks in patients with moderate-to-severe Crohn’s disease [15]. Statistical significance values refer to those reported in the original papers.

Three double-blinded multicentric randomized studies (UNITI 1, UNITI 2, IM UNITI) first evaluated the efficacy and safety of ustekinumab in CD [15,16] (Figure 1 and Figure 2).

The induction trials, UNITI-1 and UNITI-2, randomly assigned patients with moderate-to-severe active CD to the following three treatment arms: UST 130 mg, UST 6 mg per kilogram of body weight or placebo [15]. The UNITI-1 trial included 741 patients who did not respond or lost response to anti-TNFα, or experienced side effects to these drugs. The UNITI-2 trial included 628 patients who failed to achieve remission with conventional therapy; they could have previously received one or more TNF antagonists.

Both UNITI-1 and UNITI-2 showed significantly higher rates of clinical response after 6 weeks in CD patients receiving a single intravenous dose of UST compared with placebo (UNITI-1: 34.3% for UST 130 mg, 33.7% for UST 6 mg per kilogram and 21.5% for placebo, *p* ≤ 0.003 for both therapeutic regimens vs. placebo; in UNITI-2: 51.7% for UST 130 mg, 55.5% for UST 6 mg per kilogram and 28.7% placebo, *p* < 0.001 for both therapeutic regimens vs. placebo).

All major and minor secondary endpoints, including the reduction in C-reactive protein (CRP) or fecal calprotectin (FC) levels, documented a significant advantage of the two UST-treated groups compared to placebo.

The maintenance study, IM-UNITI, involved 388 responders to induction trials UNITI-1 and UNITI-2, randomly assigned to SC UST, 90 mg every 12 or 8 weeks versus placebo, for 44 weeks [16]. The primary endpoint was clinical remission at week 44 (Crohn’s disease activity index, CDAI score < 150). The major secondary endpoints at week 44 were clinical response (defined as a reduction from baseline in the CDAI score of greater than or equal to 100 points) and steroid-free remission. The trial documented the efficacy of SC maintenance treatment, every 8 or 12 weeks, versus placebo in maintaining clinical response at week 44. Efficacy and exposure–response data favored the administration every 8 weeks compared to every 12 weeks.

Patients who responded to SC therapy were subsequently included in a long-term extension trial, which showed that SC UST was well tolerated and proved effective in maintaining clinical remission through 5 years (61.9% for patients q12w and 69.5% for q8w). Long-term safety profile and immunogenicity were also encouraging, and similar to those observed in induction and maintenance trials.

A recent metanalysis of 38 observational studies reported pooled clinical remission rates for CD of 34% after the induction and 31% at 1 year [17].

Limited data concern the efficacy of UST for the treatment of extraintestinal manifestations (EIMs). A post hoc analysis of UNITI-1/2 and IM-UNITI suggested that UST was not superior to placebo in inducing a remission rate of EIMS at weeks 6 and 52 (36.9% UST and 39.1% placebo, *p* = 0.564 after 6 weeks; 76.4% UST vs. placebo 80%, *p* = 0.524 after 52 w) [18].

### 3.2. Ustekinumab versus Other Therapies in CD

A post hoc analysis of two clinical trials (CT-P13 study and UNITI-2 study) compared the efficacy of induction with infliximab (IFX) versus UST in 420 moderate-to-severe, biologic-naïve, CD patients, with comparable results. Clinical remission with IFX or UST at week 6 were 44.9% and 37.9%, respectively. The clinical response rate at week 6 was about the same (58.4% vs. 54.9%) [18].

A comprehensive comparison of UST or adalimumab (ADA) in the United States has been provided by a large real-world retrospective study in biologic-naïve CD patients. Five thousand ninety-one CD patients were enrolled (948 UST and 4143 ADA). After 14.9 months follow-up in the UST cohort and 16.9 months in the ADA cohort, UST had a 50% higher rate of treatment persistence compared to ADA (HR: 1.50; 95% CI: 1.29–1.74; *p*-value < 0.001). The UST cohort had a 17% higher rate of corticosteroid-free remission compared to ADA (HR: 1.17; 95% CI; 1.04–1.31; *p*-value = 0.008). Within 12 months of maintenance, 11.2% of patients in the UST- and 16.9% of the ADA-treated cohort required dose escalation. These findings suggested that UST could represent an effective first-line treatment in CD [19].

Conversely, the SEAVUE trial, including 386 naïve CD patients randomly assigned to UST or ADA, after 1 year of follow-up could not document significant differences in efficacy (clinical remission: 65% UST vs. 61% ADA, *p* = 0.4) and safety (severe infections: 2% UST vs. 3% ADA [20].

A retrospective study based on the Korean National Database analyzing treatment persistence in 2987 CD patients who were starting biologics suggested that UST is superior to anti-TNFα in bio-naïve subjects [21].

Moreover, in a smaller study (97 ADA and 66 UST), the clinical response rate (73.2% vs. 50%, *p* < 0.02) and the remission rate (44.3% vs. 27.7%, *p* < 0.032 at 16 weeks) were higher in the ADA group. A nonsignificant trend favoring UST in the clinical response rate (52% vs. 25%, *p* < 0.24) and the clinical remission rate (25% vs. 27%, *p* < 0.82) was observed, as expected, in the subgroup of anti-TNFα–experienced patients [22].

The efficacy of UST versus a second anti-TNFα agent in anti-TNFα–experienced patients was evaluated in a retrospective cohort. Treatment persistence at 3 years were similar (35% of UST vs. 36% of anti-TNFα, *p* = 0.72). No significant difference was observed in the 3-year hospital admission rate (28% vs. 30%, *p* = 0.99), infection-related hospital admission (8% vs. 8%, *p* = 0.31), surgery (13% vs. 8%, *p* = 0.17) and a need for antibiotics (51% vs. 50%, *p* = 0.56). The proportion of patients still using the second-line therapy was not influenced by the first-line anti-TNFα (ADA or IFX) [23].

Instead, no difference in clinical remission rates was observed between anti-TNFα and UST (48.3% vs. 56%, *p* = 0.8) as second-line biological therapy after VDZ failure [24].

UST and Vedolizumab (VDZ) were compared in a cohort of 470 anti-TNFα–experienced CD patients [25]. Similar clinical remission rates were reported at 26 weeks (UST 60.1% vs. VDZ 65.4%, *p* = 0.277), but VDZ was associated with higher remission rates at 1 year (55.5% vs. 42.5% UST, *p* = 0.038). Conversely, in a study carried out in anti-TNFα–experienced CD patients (107 UST and 132 VDZ), UST proved more effective at week 48 in inducing clinical remission (54.4% vs. 38.3%; odds ratio (OR) = 1.92, 95% CI [1.09–3.39]), more so in ileal and penetrating disease. Treatment persistence was also better in the UST arm (71.5% vs. 49.7%; OR = 2.54, 95% CI [1.40–4.62], but not the steroid-free clinical remission rate (44.7% vs. 34.0%; OR = 1.57, 95% CI [0.88–2.79]) [26].

Following the failure an anti-TNF agent in 203 CD patients, UST, VDZ or a second anti-TNF agent showed similar steroid-free remission rates (29%, 38% and 44%, respectively, *p* = 0.15) at weeks 14 and 24. After a mean follow-up of 118 weeks (±93), the drug persistence was shorter in patients who received UST (*p* = 0.001) [27]. A smaller cohort confirmed these results [28]. The clinical remission rates were similar in UST- and VDZ-treated patients (CDAI < 150, 50.0% vs. 53.6%, *p* = 0.820) as well as sustained clinical response at week 48 (*p* = 0.692) and safety profiles. Nonetheless, the size of these cohorts prevents definitive conclusions.

Recently, UST was compared to guselkumab (GUS) in patients completing the 48-week maintenance therapy with GUS in the GALAXI-1 long-term extension (LTE) study. As expected for long-term responders, endoscopic response at week 144, defined as ≥50% improvement from baseline in the simple endoscopic score for CD (SES-CD) or SES-CD ≤ 2, was superior in the GUS arm as compared to UST (34.7% vs. 19.4%) [29]. However, a post hoc analysis of the same trial suggested a higher endoscopic response compared to UST also in patients who did not achieve a 12-week clinical response (31.7% and 23.8%, respectively) [30].

The efficacy of UST was also compared with Risankizumab, with the latter showing higher rates of endoscopic remission at week 48 (16.2% vs. 31.8%) [31].

UST proved superior to azathioprine in preventing endoscopic postoperative recurrence (POR) in a retrospective study involving 63 CD patients. Endoscopic POR (Rutgeerts’ index ≥ i2) and severe endoscopic POR (Rutgeerts’ index ≥ i3) were 20.8% versus 42.5% (*p* = 0.066) and 16.9% versus 27.9% (*p* = 0.24) following UST and azathioprine, respectively [32].

A similar risk of overall postoperative complications was observed in 30 CD patients treated with UST and 73 with VDZ at week 12 (OR 95% CI 0.38 (0.10–1.4); *p* = 0.15) [33].

Endoscopic recurrence rates within a year following surgery (Rutgeerts score ≥ i2) were also compared in patients undergoing ADA or UST. Despite UST patients being older, having longer disease duration and being exposed to more biologics, endoscopic recurrence rates were nonsignificantly higher (58.3% vs. 47.6%, *p* = 0.3) [34].

### 3.3. Dose Escalation and Reinduction in CD

Like with other biologics, a proportion of patients experience primary or secondary nonresponse to UST, and dose escalation has been considered. Despite the wide use of UST in clinical practice, relatively few data are available on dose optimization or reinduction in this subset of patients.

#### 3.3.1. Dose Escalation and Shortening the Interval between Doses

As previously reported, the IM-UNITI trial favored the administration every 8 weeks over longer intervals (Figure 2) [15].

Similar results have been reported in a cohort of 139 CD patients allocated to UST maintenance every 8 weeks (85 patients) or 12 weeks (54 patients) [35]. This series included a higher percentage of anti-TNF-α and anti-integrin–exposed patients than the IM-UNITI trial, and may be considered more representative of real-life clinical practice. A shortened administration interval correlated with lower discontinuation rates (20% vs. 42.6%, *p* = 0.01) at week 52, but not with corticosteroid-free clinical remission (46.3% vs. 34.6%, *p* = 0.20). Despite similar baseline characteristics, the Harvey–Bradshaw Index (HBI) was significantly higher at week 12 in the q8w-treated group (6 vs. 5, *p* = 0.04), suggesting that the 8-week interval was preferred in patients with more aggressive disease [35]. This may represent a relevant bias and affect conclusions.

Several studies suggest the opportunity to further shorten the intervals to 6 or 4 weeks (Table 1, study design reported in Supplementary Table S1) [36,37,38,39,40,41,42,43,44,45,46,47,48,49,50].

The drug administration interval was shortened from 8 to 4 weeks in 110 nonresponders out of 506 CD patients after a median time of 7.5 months. Following dose escalation, clinical remission (HBI < 4) was observed in 50.9% of patients, after a median time of 5 months. A reduction in CRP < 5 mg/L and an improvement of FC (FC < 250 μg/g) were observed in 29.1% and 50% of patients, respectively. Endoscopic remission (quiescent disease) was reported in 36% of patients who had baseline moderate-to-severe disease [36].

Similarly, a retrospective observational study proved that a 4-week dose escalation improves clinical symptoms evaluated by the physician global assessment score (PGA) (0.47 ± 0.19, *p* < 0.05) and reduces CRP (0.33 ± 0.19 mg/L, *p* < 0.05), but also improves albumin levels (0.23 ± 0.06 g/dL, *p* < 0.05) [37].

Dose intensification to 4 and 6 weeks was retrospectively evaluated in 123 patients in a study comparing the efficacy of the two regimens. The study reported that both strategies were clinically effective, because more than 50% of patients in the two groups achieved corticosteroid-free remission at 12 and 24 months. Interestingly enough, more than 80% of CD patients who were in corticosteroid-free remission at week 12 maintained remission at week 24. However, q4w patients had a significantly higher need for systemic corticosteroids (31.3% vs. 14.6%, *p* = 0.03) and longer CD duration at baseline (15.5 years vs. 12.7, *p* = 0.08). As in other retrospective studies, q4w patients likely had more severe disease, and the authors concluded that similar efficacy of the two regimens could not be unequivocally supported by their data. As secondary outcomes, the study suggested that, as expected, perianal disease, a higher HBI and use of opioids and corticosteroids were associated with UST failure [38].

Fumery et al. showed that after a median of 2.4 months after dose intensification (4 weeks) clinical response and clinical remission were observed, respectively, in 61% and 31% of patients who lost response to q8w administration [39].

Other studies in smaller series confirmed that shortening the administration interval from 8 to 4 weeks was effective and safe in regaining response and inducing clinical remission, with the median time varying from 4 weeks to 6 months (Table 1) [36,37,38,39,40,41,42,43,44,45,46,47,48,49,50].

According to Hanzel, in 44 patients who underwent a 4-week dose escalation, endoscopic remission (SES-CD ≤ 3 without ulceration) was achieved in 28.6%, CRP normalization in 29.2% and FC normalization in 51.7% of patients [40].

Similarly, UST dose escalation to 90 mg every 4 or 6 weeks led to clinical remission within 3–6 months in 30.9% of the 68 patients. Of the patients with available endoscopic follow-up data (n = 39), 59% had endoscopic response and 20.5% achieved endoscopic remission [41].

Overall, the endoscopic remission rate following a shortened administration interval varied in differing series from 8.6% to 43.4%, and endoscopic improvement was observed in about two-thirds of patients (Table 1) [36,37,38,39,40,41,42,43,44,45,46,47,48,49,50]. A recent metanalysis including 126 patients reported endoscopic improvement in 61% (CI 52–69%) of patients and endoscopic remission in 29% (CI 16–44%) [51].

The q4w administration was more effective in preventing recurrence compared to the 8-week regimen (*p* < 0.05) [37].

Data on the efficacy of UST dose escalation on perianal disease have been reported in some studies.

Twelve out of twenty-four patients with perianal disease included in a series of thirty-eight patients who did not respond to standard UST maintenance and underwent dose escalation to 90 mg every 4 or 6 weeks showed improvement and few patients required surgery [42].

In another series of 28 patients with perianal disease, 36% achieved clinical remission of perianal fistulas at week 24. No difference was found between the q8w and q12w treatment schedule [35]. Resolution of perianal symptoms was reported in 5 of 11 patients (45%) [36].

These reports are encouraging but no clear-cut conclusions may be drawn because the efficacy of a reduced UST administration interval on perianal disease results from small, retrospective series.

#### 3.3.2. Intravenous Reinduction in CD Patients who Lost Response to UST or with an Unsatisfactory Response to “Conventional” Dose Escalation

The efficacy and safety of single IV reinduction (6 mg/kg) followed by SC 90 mg UST q8w versus standard 90 mg SC UST q8w was evaluated in 215 CD patients with secondary loss of response to UST 90 mg q8w. A higher proportion of patients from the IV arm achieved clinical response at week 16 (49.1% vs. 37.4%, *p* = 0.089). Endoscopic remission, FC normalization and IBDQ-score improvement also favored the IV arm (18.6% vs. 5%, *p* = 0.043; 17% vs. 9%, *p* = 0.145; 59% vs. 43%, *p* = 0.017, respectively). It thus appears that a single IV administration significantly improves standard q8w maintenance therapy [44]. Recently, a post hoc analysis of this study suggested that patients with a higher disease burden at baseline (disease extent or elevated inflammatory biomarkers) were more likely to benefit from UST IV reinduction [52].

Dose escalation (4 or 6 weeks), intravenous reinduction or a combination of the two were retrospectively compared in a multicenter cohort of 142 CD patients. Ninety-one (64.1%) of the patients were escalated to the q4w regimen, twenty (14.1%) patients were escalated to q6w, fourteen (9.9%) patients received an IV reinduction and seventeen (12%) patients received a combination of IV reinduction and interval shortening. Clinical response at week 16 (ΔHBI ≥ 3 or ΔCDAI ≥ 70) was achieved by 51.4% of patients, CRP normalized in 21.4% of patients and corticosteroid-free remission was achieved by 17.6% of patients on corticosteroids upon escalation. The likelihood of achieving clinical response at week 16 did not statistically differ in patients who received intravenous reinduction versus those who received SC interval shortening (65.8% with q4w, 13.7% with q6w, 13.7% with q4w and 6.8% with IV reinduction, *p* = 0.48). Thus, nonresponders may benefit from dose escalation, but no differences between SC escalation and IV reinduction were detected [45].

Similar results were reported in a smaller study [53].

A multicenter, retrospective cohort study involving 56 CD patients reported the efficacy of IV UST reinduction after a partial response or loss of response on SC UST maintenance. Clinical remission at week 16 was observed in 43.3% of patients undergoing IV reinduction, as well as an improvement in biomarker values (PCR 6.3 ± 7.4 mg/L, *p* = 0.08; FC 194 ± 154 μg/g, *p* = 0.22) [46].

#### 3.3.3. IV Ustekinumab at Regular Intervals in CD

This issue was addressed in a retrospective study in 79 patients (73 CD and 6 UC) undergoing IV doses every 4–6 weeks following an inadequate response or loss of response to 90 mg SC every 4–6 weeks. Twelve weeks after the first IV dose, 43% of patients were in clinical remission (HBI < 5). The figure increased to 59.5% after a 14-month follow-up. Basal FC significantly decreased at month 12 and at the end of follow-up (612.6 mg/kg vs., 384.1 mg/kg vs., 222 mg/kg; *p* = 0.0002 and *p* = 0.0048, respectively). Thus, prolonged intravenous maintenance treatment proved effective in a large proportion of patients failing to respond to intensified SC UST [47].

Similarly, a recent small Spanish study confirmed these results, because UST 130 mg every 4 weeks induced clinical and biochemical improvement in 27 CD patients [54].

While dose escalation and reinduction both improve clinical and endoscopic outcomes in CD, it is still unclear how to identify those patients who will profit from more aggressive therapeutic strategies.

Baseline CPR was reported by a Finnish nationwide real-world study as the only effective predictor of patients requiring dose intensification [55]. Fistulizing behavior, ≥2 biological failure and lack of steroid-free remission at week 12 after UST induction all predicted poor efficacy of standard dose escalation in an Australian retrospective study [56]. This was well expected because these parameters identify a subset of patients with particularly aggressive and resistant disease. In another study, response to the UST induction dose was the best predictor of subsequent response to UST dose escalation (OR: 7.01; 95% CI 1.83–27.07; *p* = 0.005) [41]. This, again, could be anticipated.

Adverse events following escalation were reported in 11 (7.7%) of the 142 patients who underwent dose escalation every 4 or 6 weeks in the Kopylov series. These events were usually mild, consisting of skin eruptions in two patients, acute gastroenteritis of unknown etiology in two patients and one case each of a clostridium difficile infection, CMV colitis, concentration disturbance and a benign breast lump. A few severe adverse events were also reported in the series consisting of cervical intraepithelial neoplasia grade 1, nonmelanoma skin cancer and an upper respiratory tract infection that required hospitalization, one case each [45].

No serious adverse events were reported in 65 patients who underwent UST reinduction. One patient experienced facial erythema and mild dyspnea but completed the infusion at a lower rate [49].

Thus, UST dose escalation and reinduction are safe and should be considered before switching out of class. However further studies are required to compare the two strategies and define the optimal therapeutic administration intervals and evaluate long-term safety of these approaches.

### 3.4. Ustekinumab, Registrative Trials and Treatment in Naïve UC Patients

**Figure 3:**
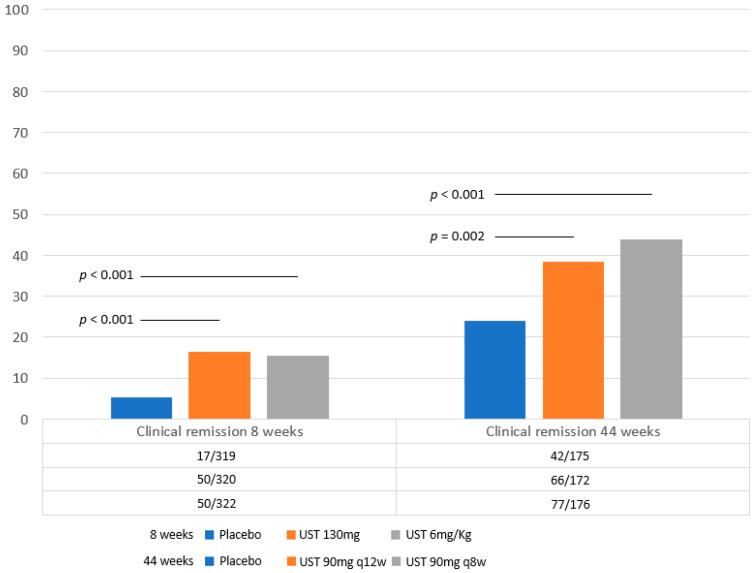
**Endpoints of the UNIFI trial.** Clinical remission rates at 8 and 44 weeks in patients with moderate-to-severe ulcerative colitis treated with Ustekinumab or placebo (UNIFI study) [57]. Statistical significance values refer to those reported in the original papers.

The efficacy and safety of induction and maintenance of UST in patients with moderate-to-severe UC were reported in the UNIFI randomized controlled trial [57]. Nine hundred sixty-one patients were randomly assigned to three induction treatment groups with intravenous UST at the dose of 130 mg or 6 mg/kg body weight, or placebo. Responders to induction therapy at 8 weeks subsequently received subcutaneously maintenance doses of 90 mg UST every 8 or 12 weeks, or placebo (Figure 3).

At week 8, the clinical remission rate was superior to placebo, but nonetheless unsatisfactory, ranging about 15%. Clinical response and endoscopic improvement (Mayo score of 0 or 1) were significantly higher following the UST 130 mg (51.3% and 26.3%) and UST 6 mg/kg groups (61.8% and 27%) than in the placebo group (31.3% and 13.8%) (*p* < 0.001).

Persistent clinical remission in responders was significantly higher at 44 weeks in the UST-treated patients q12w (38.4%) and q8w (43.8%) than with placebo (24.0%) (*p* < 0.002).

The same proved true for clinical response in patients receiving UST every 12 weeks, UST every 8 weeks or placebo-favored UST (68%, 71% and 44.6%, for q12w, q8w and placebo), endoscopic improvement (43.6%, 51.1% and 28.6%, respectively *p* < 0.001) and corticosteroid-free clinical remission (37.8%, 42% and 23.4%, *p* < 0.001).

A recent metanalysis reported a postinduction clinical remission rate of 39% [17].

The incidence of adverse events did not differ in UST- and placebo-treated patients, ranging about 45%, but mainly consisting of mild adverse events.

On the base of these results, UST treatment may be considered for the treatment of patients with moderate-to-severe UC.

### 3.5. Ustekinumab versus Other Therapies in UC

Head-to-head comparison of different biological drugs or immunosuppressants is essential for precision medicine and a patient-tailored therapeutic approach. Data, however, are scarce and largely derive from indirect comparisons or retrospective studies.

A relatively small number of studies compared the efficacy of UST versus VDZ or anti-TNFα in UC patients.

A multicentric GETAID cohort study did not find differences between UST and VDZ in terms of steroid-free clinical remission at week 52 in anti-TNFα–exposed patients (OR = 0.55 [0.21–1.41], *p* = 0.21 and 0.94 [0.40–2.22], *p* = 0.89) [58].

Despite no difference was observed in terms of clinical remission, UST showed a higher efficacy in terms of endoscopic remission at week 16 (17.5% vs. 5.3%; OR = 3.77 [1.25–11.36]; *p* = 0.018) [59].

UST, anti-TNFα and VDZ were prospectively evaluated in 317 UC patients. The size of the treatment groups was comparable (101 patients UST, 106 anti-TNFα -ADA 24.5%, IFX 65.1%, Golimumab 10.4% and 110 VDZ). At week 16, the efficacy of UST was comparable to that of anti-TNFα and VDZ in terms of clinical response (UST 36.7%, anti-TNFα 46.8%, VDZ 45.9%; *p* = 0.292), clinical remission (UST 25.6%, anti-TNF 22.3%, VDZ 32.4%; *p* = 0.475) and steroid-free remission (UST 25.6%, anti-TNFα 22.3%, VDZ 30.3%, *p* = 0.401) [60].

UST was retrospectively compared to the Jak-inhibitor tofacitinib in 289 anti-TNFα–exposed adult patients. Endoscopic remission at week 16 was achieved in 17.0% and 11.7% of patients (*p* = 0.47) and histological remission (CFREM +MES ≤ 1 + Nancy index ≤ 1) in 4.4% versus 7.8% in UST- or TOF-treated patients, respectively (*p* = 0.32) [61]. However, the authors suggested that compared to TOF, the efficacy of UST could be more impacted by prior therapeutic failures [62].

Similar results were reported in 81 UC patients (45 TOF and 34 UST) following anti-TNFα and anti-integrin failure. Steroid-free clinical remission was similar at week 12/16 (43.9% TOF vs. 40.0% UST; *p* = 0.82). Drug discontinuation rates or colectomy occurred in 51.1% of patients in the TOF group and 36.1% of patients in the UST group. The occurrence of adverse events was not significantly different (11.1% TOF vs. 5.6% UST, *p* = 0.57) [63].

Overall, these studies suggest similar efficacy of UST and TOF in UC after anti-TNFα failure. However, considering the faster onset of efficacy of TOF compared to UST, studies comparing the two drugs after longer follow-up are required before drawing definitive conclusions.

In the absence of head-to-head data, a placebo-anchored, matching-adjusted, indirect comparison of the induction treatment with UPA versus UST favored UPA, with similar safety profiles, in patients with moderate-to-severe UC. Again, these data need to be confirmed [64].

### 3.6. Dose Escalation and Reinduction in UC

UST was approved for the treatment of UC in 2019 in the United States and, more recently, in Europe. Thus, less data are available on dose escalation and reinduction of UST, compared to CD, mainly deriving from mixed IBD cohorts, or abstracts of Congress communications in the absence of papers in extenso. Both dose escalation and reinduction, however, improve the efficacy of UST, compared to the standard dose interval.

The long-term extension UNIFI trial evaluated the efficacy of dose adjustment from q12w to q8w in 523 UC patients at week 56. Clinical remission at week 56 was observed in 70.0% of patients shortening the administration interval. Because most patients were already in remission at optimization, or had mild activity, the real value of these data is debatable [62].

Intensification, every 4 or 6 weeks, was required after a median time of 95 days in 42.6% of 108 UC patients [65]. Twelve to sixteen weeks after intensification, 55.0% achieved remission and 67.5% achieved response (reduction in SCCAI/Mayo by 3 points from baseline). After a median follow-up of 230 days after dose escalation, 56.3% had endoscopic improvement and FC reduction (Table 2, study design reported in Supplementary Table S1) [43,66].

This was confirmed in a small retrospective cohort of 23 patients. During the 12 months follow-up, dose adjustment was required in 13 patients (56%). Three (13%) underwent intravenous UST reinduction and ten (43%) dose escalation (eight by shortening the interval between doses and two by switching to intravenous USK administration). After 52 weeks from dose adjustment, clinical remission was reached in 79% of the patients, and normalization of CRP and FC in 80% and 40%, respectively. Five of the six patients who underwent endoscopic examination following adjustment showed a Mayo subscore ≤ 1 [67].

A real-world retrospective cohort study in 40 UC patients undergoing dose intensification q4w or q6w provides similar data. More than 50% of patients achieved corticosteroid-free clinical remission, defined as HBI < 5 and no use of systemic steroids within 30 days, at 1 year and more than 40% at month 24. Interestingly, more than 80% of the UC patients in corticosteroid-free remission at week 12 maintained remission at week 24. Endoscopic response was observed in 55% of those patients undergoing the procedure while on UST [43].

More data were derived from a small, mixed cohort of 42 IBD patients (8 UC) undergoing dose escalation q4w or q6w or dose intensification followed by IV reinduction. Fifty-two weeks later, 14 patients (33.3%) achieved corticosteroid-free clinical remission, biochemical remission and endoscopic healing. No significant advantage was provided by reinduction (*p* = 0.99) [50].

A few case reports also suggested the efficacy of dose escalation, with one case documenting clinical improvement following a q3w injection regimen. No adverse events were reported in the 6-month follow-up [68].

Despite the lack of hard data on UST dose intensification and reinduction in UC, available evidence suggests that, similarly to CD, this may represent a viable strategy in primary nonresponse and secondary loss of response. Prospective studies and larger series are needed to confirm preliminary evidence and identify specific subpopulations that would benefit from the differing optimization strategies.

## 4. Conclusions

Ustekinumab is a fully human antibody preventing the bond of the subunit p40 of IL-12 and IL-23 with the receptor proteins expressed on immune cells. It hinders IL-12 and IL-23 biological activity, affecting T-cell differentiation toward Th1 lineage and Th17 cell-related immune response [69]. First licensed for psoriasis and psoriatic arthritis, UST received regulatory approval for CD in 2016 and UC in 2019 [15,57].

UST proved effective in inducing and maintaining clinical response and remission in IBD, and is presently considered a viable second-line treatment. Head-to-head comparisons of different biologics are scarce, and evaluations mostly derive from indirect comparison or retrospective studies. Available evidence suggests that anti-TNFs are superior to UST as first-line therapy in CD. The efficacy of UST as second-line therapy ranges between 35% and 50% [23,24,25,27,28]. Figures are comparable to other second-line therapies in terms of the response, remission and adverse event rates in uncomplicated CD [70,71]. Efficacy is comparable to other biologics also in fistulizing CD [68] while experience is poor in postoperative recurrence.

Less data are available in UC patients compared to CD, but no major differences have been reported versus other second-line biologic agents [58,60]. Network metanalysis reported similar efficacy of UST and TOF in UC patients, both proving superior to VDZ [72]. Again, conclusions were drawn on the base of statistical tools, and need confirmation in real-life, head-to-head studies. Despite low or moderately-low evidence, UST was recommended, as well as TNF agents VDZ and TOF, by the 2022 ECCO consensus in patients with moderate-to-severe UC and inadequate response or intolerance to conventional therapy [73].

Like all other biologics and small molecules, UST is burdened by a proportion of primary nonresponse and loss of response during maintenance therapy. Dose escalation and reinduction strategies before switching out of class have been reported in an increasing number of studies, but the best therapeutic strategy and its positioning in the treatment workup of IBD is still undefined.

The IM-UNITI trial reported higher rates of drug persistence in the 8-week arm versus q12w in CD, but a nonsignificant trend in corticosteroid-free remission [16].

Some studies evaluated the further shortening of UST administration intervals to 6 or 4 weeks, or a second IV reinduction in patients with unsatisfactory response to “conventional” dose escalation.

Similarly, the UNIFI trial also favors q8w versus q12w administration in UC [54,62]. Due to the more recent approval by regulatory institutions, data on 6- and 4-week administration of UST in UC are less extensive. Nonetheless, data confirm similar rates to those reported in CD.

Considering the low immunogenicity of UST compared to anti-TNF agents [15], and the annual loss of response rate being 21% per patient/year in primary responders [74], the likelihood of effective dose escalation in UST is high. Response is recaptured following dose escalation in approximately 50% of CD [36,38,39] and UC patients who lost response [43,66], thus comparable to those observed with TNFα antagonists and vedolizumab. Some evidence supports the efficacy of UST IV reinduction or IV administration of the drug [44,45,46,53,54], but is still unclear whether the intravenous route of administration offers advantage over short-interval SC doses.

UST concentrations exceeding 3.3 µg/mL or 4.5 µg/mL following induction are associated with better results [69,75]. High UST trough levels are associated with more favorable results [69,75,76] and are significantly higher in patients treated with q8w than q12w, more so following dose escalation by reducing dose intervals to q6w or q4w [76]. This is expected and in keeping with the findings in patients treated with anti-TNFα and biologics interfering with leukocyte trafficking [77,78].

Long-term data on safety and drug persistence following UST dose escalation are lacking, but, all given, this is not cause of concern. Instead, the positioning of UST reinduction or dose escalation, instead of switching out of class, following loss of response strongly depends on cost evaluation. UST is indeed more expensive than traditional immunosuppressants and anti-TNFα agents, but comparable to other more recent biologics. Cost coverage by individual national health systems is variable in differing countries, as well as timing and extent of repricing politics. This, and safety considerations, represent the major factors in decision making, more so considering that the mean age of patients treated in the real-world experience is higher than that in clinical trials [79].

In conclusion, according to registrative trials and real-world evidence, UST is safe and effective in IBD. However, like all other biologics, in addition to primary nonresponders, a proportion of patients lose response. The overall annual risk of loss of response is 21% per patient/year in CD and that of dose escalation in primary responders is 25% per patient/year [74]. Data reported in the present narrative review indicate that over half of these patients may benefit from dose escalation or reinduction and recapture response without switching out of class. This evidence is supported by a number of observational and cohort studies, including a large series of patients, although it is still unclear which patients will most likely profit from more aggressive therapeutic strategies. A formal evaluation of the efficacy of UST reinduction or dose escalation, however, was not the primary outcome of randomized clinical trials. This prevented recommending this approach in the most recent international guidelines [73,80], and represents the main limitation of the present review. Another limitation resides in the use of data so far published as Congress abstracts. All considered, the off-label use of UST in the real-world experience is more frequent than expected and it seems effective and safe. UST risk stratification trials, as well as head-to-head comparisons with other biologics and small molecules, are needed to fully exploit the potential of this drug in a patient-tailored approach.

## Figures and Tables

**Table 1:** Performance of dose escalation or IV reinduction of UST in Crohn’s disease.

Author	N° Patients	Administration Interval	Follow-Up	Clinical Remission Rate	Steroid-Free Clinical Remission Rate	Endoscopic Remission Rate
Ollech 2021 [36]	110 CD	4 w	9 months (median)	50.9%	38%	36%
Haider 2020 [37]	15 CD	4 w	29 ± 8w (mean)	13.3%	-	-
Dalal 2021 [38]	42 CD	4 w	12 months	-	54.8%	63% *
Dalal 2021 [38]	33 CD	6 w	12 months	-	54.6%	63.2% *
Fumery 2021 [39]	100 CD	4 w	2.4 months (median)	31%	-	-
Fumery 2021 [39]	34 CD	4 w	6–8 months	49%	26%	35.9%
Hanžel 2021 [40]	44 CD	4 w	4.5 months			28.6%
Cohen 2019 [41]	68 CD	4 or 6 w	3–6 months	30.9%	-	20.5%
Glass 2020 [42]	38 CD	4 w (28 pts) or 6 w (10 pts)	4.5 months (median)	-	60%*	72% *
Dalal 2023 [43]	123 CD	4 w (65 pts) or 6 w (58 pts)	24 months	-	60.6%	-
Dalal 2023 [43]	123 CD	4 w (65 pts) or 6 w (58 pts)	12 months (median)	-	57.3%	43.4%
Schreiber 2023 [44]	108 CD	IV UST reinduction	16 weeks	33.3%	-	18.6%
Kopylov 2020 [45]	142 CD	4 w (91 pts) or 6 w (20 pts) or IV reinduction (17 pts)	16 weeks	38.7%	17.6%	-
Kopylov 2020 [45]	74 CD	n.r.	26 weeks (median)	42%	26.5%	8.6%
Bermejo 2021 [46]	56 CD	IV UST reinduction	8 weeks	49%	-	-
Bermejo 2021 [46]	56 CD	IV UST reinduction	16 weeks	43.3%	-	-
Garcia-Alvarado 2022 [47]	73 CD and 6 UC	IV doses every 4–6 weeks	12 weeks	43%	-	-
Garcia-Alvarado 2022 [47]	60 pts (n.r. CD vs. UC)	IV doses every 4–6 weeks	13.22 months (mean)	59.5%	-	-
Sedano 2020 [48]	15 CD	IV UST reinduction	14.9 weeks (mean)	53.3%		62.5% (5/8 pts)
Heron 2019 [49]	28 CD	IV UST reinduction	14 weeks (median)	-	53.8%	28.6%
Lim 2023 [50]	34 CD and 8 UC	4 or 6 w or dose intensification	52 weeks	-	33.3%	33.3%
Lim 2023 [50]	10 CD and 3 UC	IV reinduction	52 weeks	-	30.7%	30.7%

CD: Crohn’s disease; pts: patients; UC: ulcerative colitis; UST: ustekinumab; w: weeks; n.r.: not reported; *: improvement. For statistical significance values, refer to those reported in the original papers.

**Table 2:** Performance of dose escalation or IV reinduction of UST in ulcerative colitis.

Author	N° Patients	Administration Interval	Follow-Up	Clinical Remission Rate	Steroid-Free Clinical Remission Rate	Endoscopic Remission Rate
Dalal 2022 [66]	33 UC	IV reinduction + 4 w dose escalation	12–16 weeks	-	50%	31.25% *
Dalal 2022 [66]	13 UC	IV reinduction + 6 w dose escalation	12–16 weeks	-	66.7%	-
Dalal 2023 [43]	40 UC	4 w (26 pts) or 6 w (14 pts)	24 months	-	40%	-
Dalal 2023 [43]	40 UC	4 w (26 pts) or 6 w (14 pts)	12 months (median)	-	53%	55%

IV: intravenous; UC: ulcerative colitis; UST: ustekinumab; w: weeks; * 16 patients’ administration intervals were not reported. For statistical significance values, refer to those reported in the original papers.

## Data Availability

Not applicable.

## Supplementary Materials

The following supporting information can be downloaded at: https://www.mdpi.com/article/10.3390/jcm13143993/s1, Table S1: reports the study design of the studies on unconventional dose escalation or IV reinduction/treatment.
