# Supplementary material for: IBD Patients with Primary or Secondary Nonresponse to Ustekinumab Benefit from Dose Escalation or Reinduction
Source: J Clin Med. 2024 Jul 9;13(14):3993. doi: 10.3390/jcm13143993 (PMC11277193; doi:10.3390/jcm13143993)
Supplement: Supplementary file 1 [file jcm-13-03993-s001.zip › jcm-2979688-supplementary.pdf]

Supplementary Table S1. Characteristics of the studies on unconventional dose escalation or IV reinduction/treatment.

Author	Study design		Published in form of
Ollech 2021 [36]	Cohort study	Retrospective	Full paper
Haider 2020 [37]	Cohort study	Retrospective	Full paper
Dalal 2021 [38]	Cohort study	Retrospective	Full paper
Fumery 2021 [39]	Cohort study	Retrospective	Full paper
Hanžel 2021 [40]	Cohort study	Prospective	Full paper
Cohen 2019 [41]	Cohort study	Retrospective	Abstract
Glass 2020 [42]	Cohort study	Retrospective	Abstract
Dalal 2023 [43]	Cohort study	Retrospective	Full paper
Schreiber 2023 [44]	Randomized trial	Prospective	Abstract
Kopylov 2020 [45]	Cohort study	Retrospective	Full paper
Bermejo 2021 [46]	Cohort study	Retrospective	Full paper
Garcia-Alvarado 2022 [47]	Cohort study	Retrospective	Abstract
Sedano 2020 [48]	Cohort study	Retrospective	Full paper
Heron 2019 [49]	Cohort study	Retrospective	Abstract
Lim 2023 [50]	Cohort study	Retrospective	Abstract
Meserve 2022 [51]	Case series	Retrospective	Letter to the editor
Marín-Jiménez 2023 [52]	Randomized trial	Prospective	Abstract
Truyens 2019 [53]	Cohort study	Retrospective	Abstract
Suarez Ferrer 2024 [54]	Cohort study	Retrospective	Full Paper
Sipponen 2021 [55]	Cohort study	Retrospective	Full paper
Ramaswamy 2020 [56]	Cohort study	Retrospective	Abstract
Dalal 2022 [66]	Cohort study	Retrospective	Full paper
Iborra 2022 [67]	Cohort study	Prospective	Abstract
Chatand 2020 [68]	Case series	Retrospective	Letter to the editor
